# Differentiation of pheochromocytoma and adrenal lipoid adenoma by radiomics: are enhanced CT scanning images necessary?

**DOI:** 10.3389/fonc.2024.1339671

**Published:** 2024-09-11

**Authors:** Shi he Liu, Pei Nie, Shun li Liu, Dapeng Hao, Juntao Zhang, Rui Sun, Zhi tao Yang, Chuan yu Zhang, Qing Fu

**Affiliations:** ^1^ Department of Radiology, The Affiliated Hospital of Qingdao University, Qingdao, China; ^2^ GE Healthcare, PDx GMS Advanced Analytics, Shanghai, China

**Keywords:** adrenal adenoma, pheochromocytoma, CT, radiomics, classification

## Abstract

**Purpose:**

To establish various radiomics models based on conventional CT scan images and enhanced CT images, explore their value in the classification of pheochromocytoma (PHEO) and lipid-poor adrenal adenoma (LPA) and screen the most parsimonious and efficient model

**Methods:**

The clinical and imaging data of 332 patients (352 lesions) with PHEO or LPA confirmed by surgical pathology in the Affiliated Hospital of Qingdao University were retrospectively analyzed. The region of interest (ROI) on conventional and enhanced CT images was delineated using ITK-SNAP software. Different radiomics signatures were constructed from the radiomics features extracted from conventional and enhanced CT images, and a radiomics score (Rad score) was calculated. A clinical model was established using demographic features and CT findings, while radiomics nomograms were established using multiple logistic regression analysis.The predictive efficiency of different models was evaluated using the area under curve (AUC) and receiver operating characteristic (ROC) curve. The Delong test was used to evaluate whether there were statistical differences in predictive efficiency between different models.

**Results:**

The radiomics signature based on conventional CT images showed AUCs of 0.97 (training cohort, 95% CI: 0.95∼1.00) and 0.97 (validation cohort, 95% CI: 0.92∼1.00). The AUCs of the nomogram model based on conventional scan CT images and enhanced CT images in the training cohort and the validation cohort were 0.97 (95% CI: 0.95∼1.00) and 0.97 (95% CI: 0.94~1.00) and 0.98 (95% CI: 0.97∼1.00) and 0.97 (95% CI: 0.94∼1.00), respectively. The prediction efficiency of models based on enhanced CT images was slightly higher than that of models based on conventional CT images, but these differences were statistically insignificant(P>0.05).

**Conclusions:**

CT-based radiomics signatures and radiomics nomograms can be used to predict and identify PHEO and LPA. The model established based on conventional CT images has great identification and prediction efficiency, and it can also enable patients to avoid harm from radiation and contrast agents caused by the need for further enhancement scanning in traditional image examinations.

## Introduction

Adrenal adenoma accounts for 75% to 80% of all benign adrenal tumors and is the most common adrenal tumor (1). The clinical symptoms are often nonspecific. Depending on the lipid content of the tumor, a CT value of 10 HU is taken as the boundary. If the average CT value within the lesion is lower than 10HU, it indicates that the lesion is an adrenal adenoma rich in lipids. If the CT value is higher than 10HU, it indicates that the lesion is an adrenal adenoma lacking in lipids (LPA) (2–5). A pheochromocytoma (PHEO), which originates from the adrenal medulla, can secrete catecholamines and cause hypertension and may lead to neuropathy and heart disease (6). When the clinical and imaging manifestations of PHEO and adrenal adenoma are not typical, the classification of the two diseases is difficult, and the misdiagnosis rate is high before surgery (7–9). Moreover, there are significant differences in preoperative preparation, surgical approach and prognosis between the two adrenal adenomas (10). Therefore, it is very important to correctly distinguish these two diseases before surgery.

The aim of our research is to develop different radiomics models based on conventional CT scan images and enhanced CT images to identify PHEOs and LPAs and to compare the predictive efficacy of various models to screen the most parsimonious and efficient model.

## Materials and methods

### Patients

**Figure 1:**
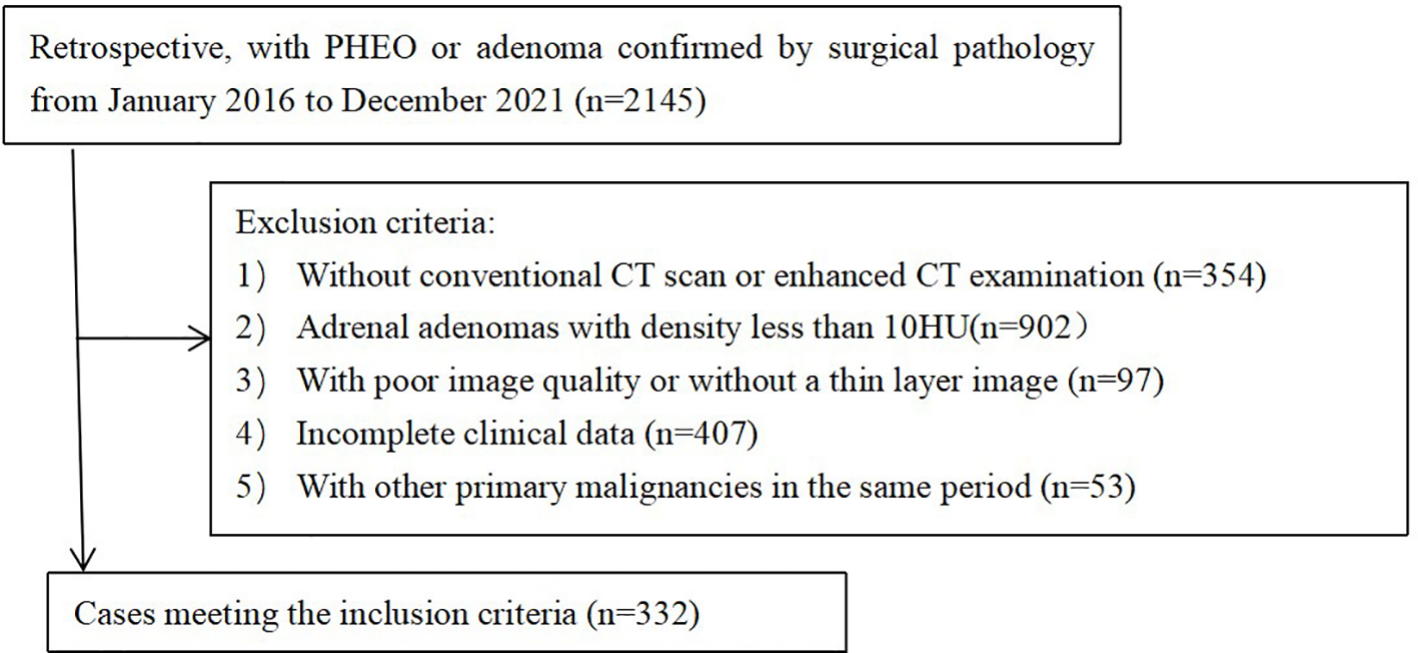
Flow diagram of the patient selection.

The imaging and clinical data of 167 patients (168 lesions) with LPA and 165 patients (184 lesions) with PHEO confirmed by surgical pathology in the Affiliated Hospital of Qingdao University from January 2016 to December 2021 were retrospectively collected (**Figure 1**). The inclusion criteria were as follows: (1) Both conventional CT scans and dynamic enhanced CT scans were performed before surgery. (2) The tumor lesions were confirmed by surgery and complete pathological data. The exclusion criteria were as follows: (1) The patient was accompanied by other primary malignant tumors during the same period; (2) The average CT number of adrenal adenoma was less than 10HU; (3) The quality of the image could not meet the requirements of analysis; and (4) With incomplete clinical data. Using a stratified random sampling method, patients were divided into a training cohort (n=232) and a validation cohort (n=100) in a 7:3 ratio.

### Image acquisition and segmentation of lesions

All CT scans were performed on one of the following devices: GE Discovery CT 750 HD (GE Healthcare, USA),SOMATOM Definition AS(Siemens Medical Systems, Germany) and Brilliance iCT (Philips Healthcare, Netherlands). The acquisition and reconstruction parameters are shown in **Table 1**. The slice thickness of the conventional CT was set at 5 mm. During the enhanceme nt scan, 50 ml iohexol (300 mg/mL) was injected into the elbow vein with a flow rate of 2.5-3.0 ml/s. The arterial and venous phase images were collected at 25 s and 65 s after the injection of contrast agent. The slice thickness of the reconstructed image was set to 1 mm.

**Table 1:** The scanning parameters and reconstruction parameters of these three CT scanners.

Parameters	Discovery 750 HD	SOMATOM Definition AS	Brilliance iCT
Scan parameters	120kVp,Smart mA	Care kV,Care Dose 4D, Ref mAs	120kVp,Dose Right
Pitch Reconstruction slice thickness(mm)	0.984 1.25	0.6 1	0.984 1
Reconstruction kernel	soft	B30f	standard

Using image segmentation software (ITK-SNAP, http://www.itksnap.org, Version: 3.8.0, USA), we manually delineated the region of interest (ROI) on the axial image that displayed the largest cross-sectional area of the lesion on the conventional CT scan. On the axial section images of the arterial phase and venous phase (with a thickness of 1 mm) of the dynamic enhanced CT scan, drew ROI layer by layer along the edge of the lesion, and then apply automatic fusion software to generate 3D ROI of the lesion. All ROI delineation was completed by 2 radiologists (Doctor QF and Doctor SLL) with more than 10 years of chest CT diagnosis experience. Dr. QF outlined the ROI and performed feature extraction. After 1 week, the second ROI mapping and feature extraction were performed to evaluate the internal consistency of the measurers. Dr. SLL only performed ROI placement and feature extraction once. This approach was used to evaluate the inter- and intra- class correlation coefficients (ICCs). An ICC > 0.75 was regarded as satisfactory inter- and intra-reader reproducibility.

### Image standardization, feature extraction and development of the radiomics signature

**Figure 2:**
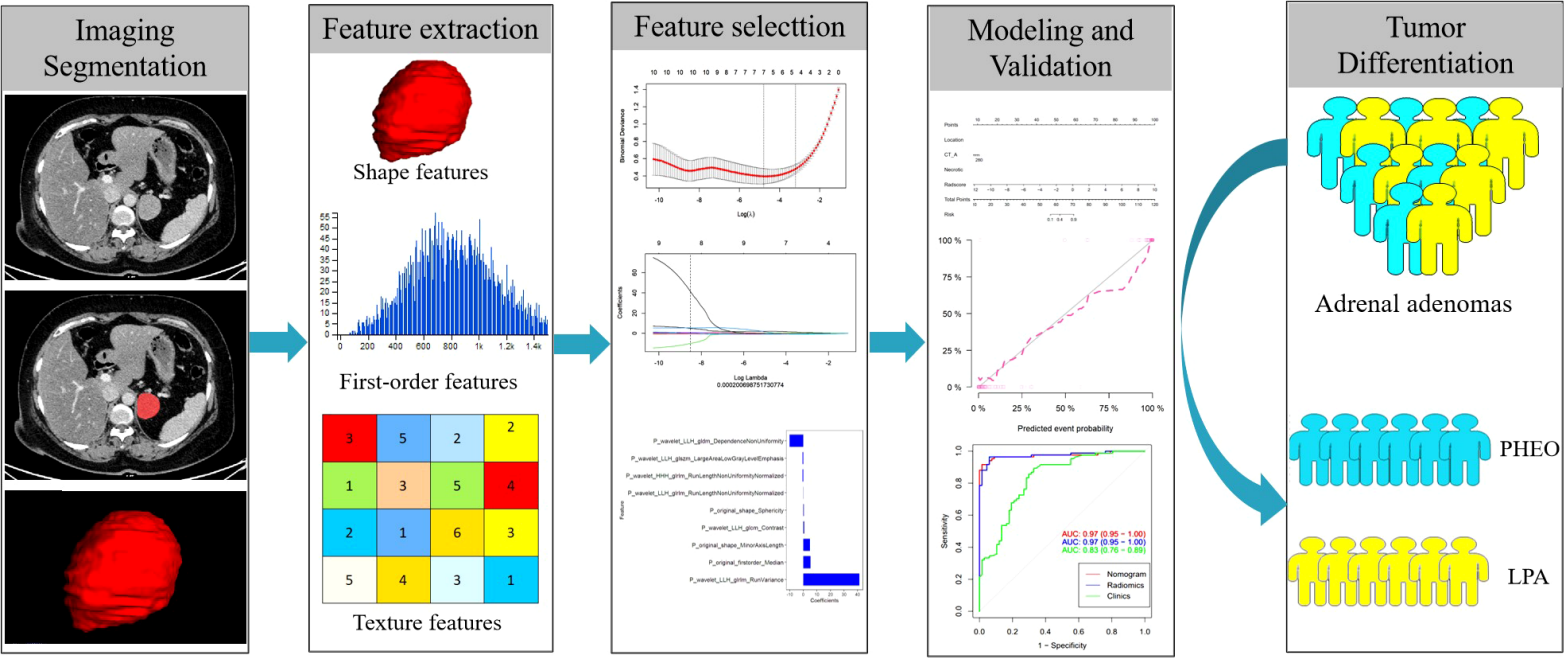
Flowchart of radiomics implementation in this study.

**Figure 3:**
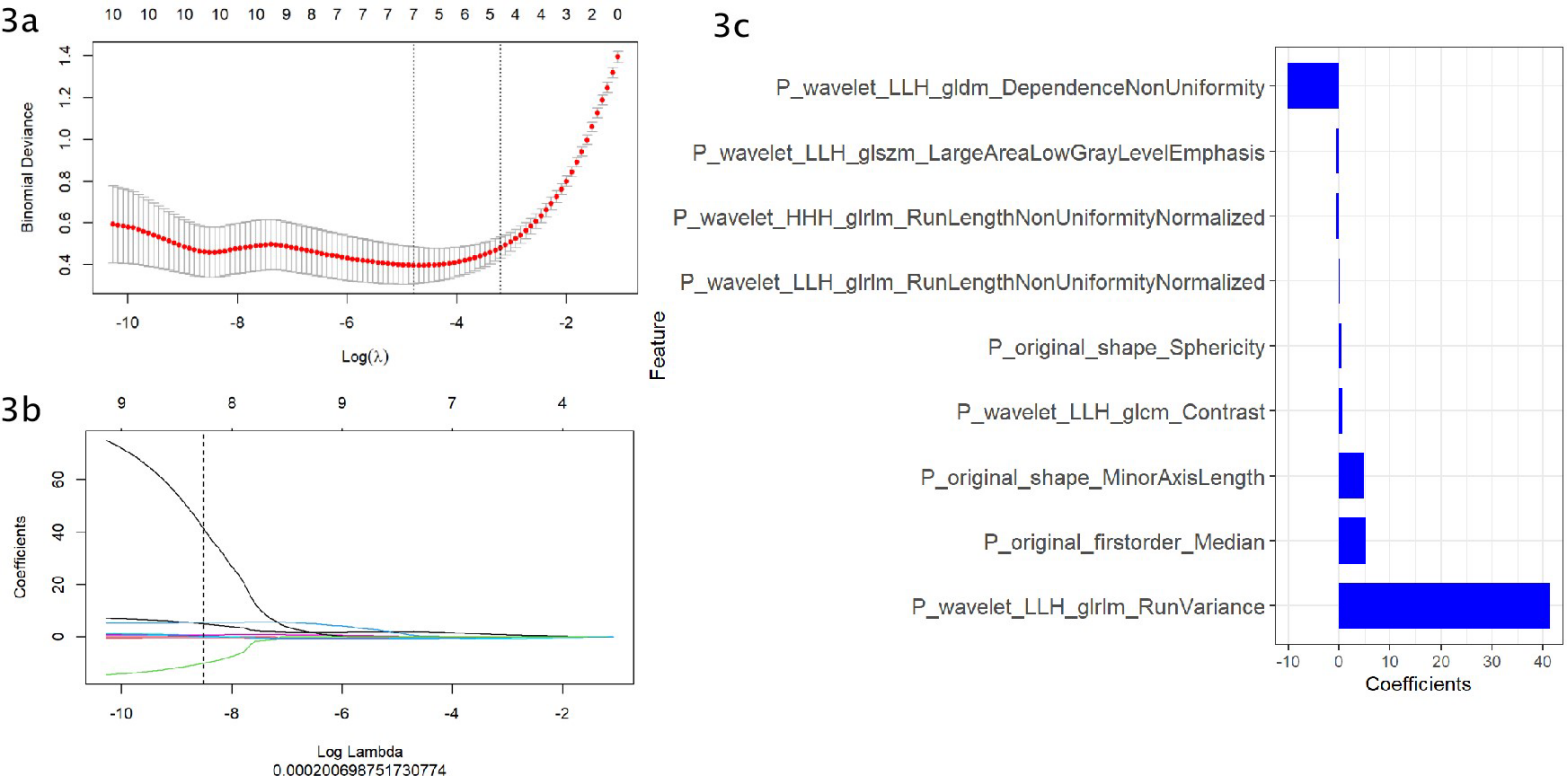
**(A–C)** Use the Least Absolute Shrinkage and Selection Operator (LASSO) regression model for radiomics feature selection. **(A)** Using cross validation to select the optimal model parameters λ. **(B)** Using 10 cross validation tests, a coefficient profile was generated and matched with the selected logarithm λ. **(C)** Nine radiomics features with nonzero coefficients were selected.

**Figure 4:**
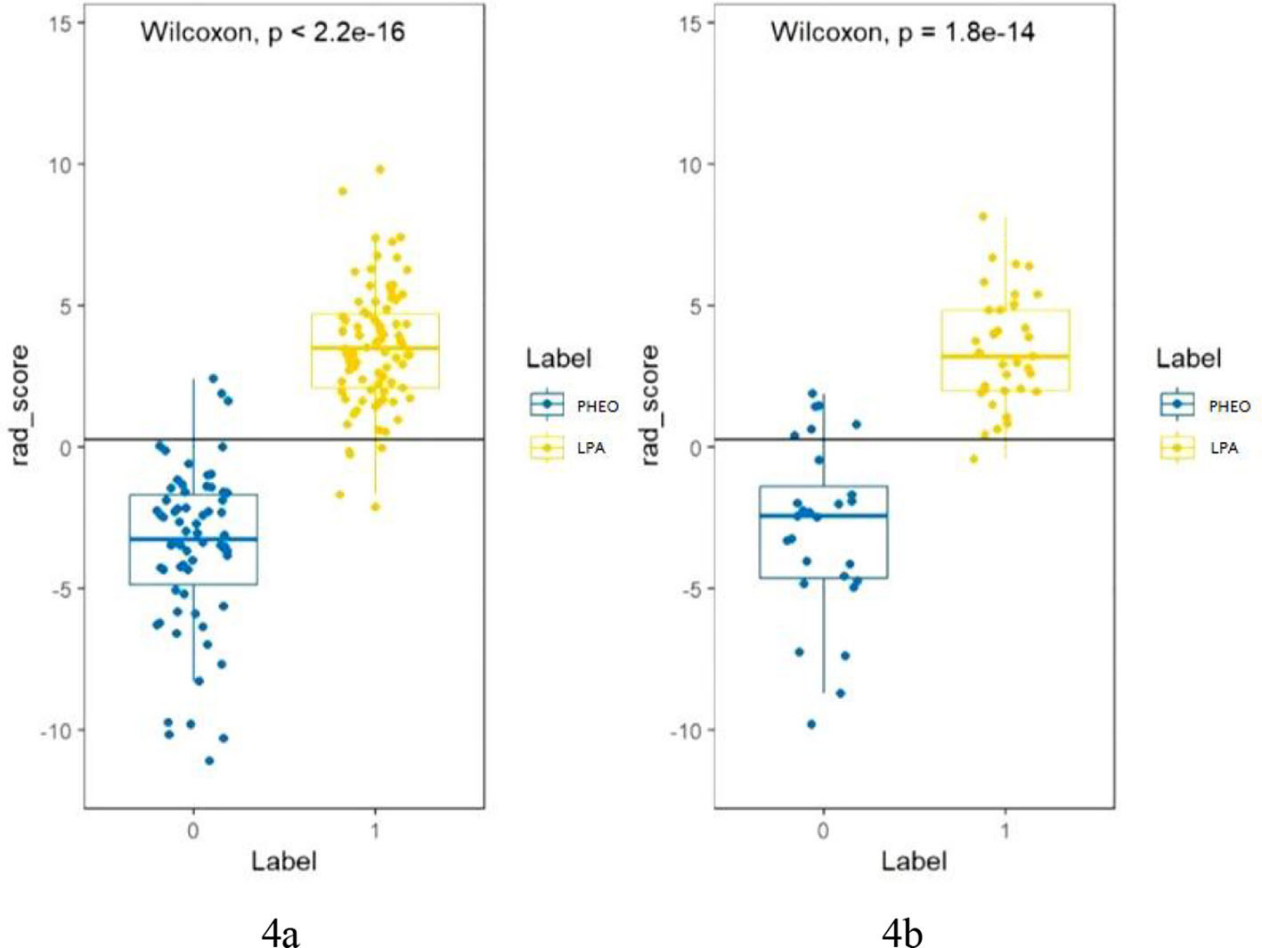
**(A, B)** shows the Rad score of each patient in the training cohort **(A)** and validation cohort **(B)**. The Rad score was used to classify patients with PHEO and LPA according to the threshold. Use Wilcoxon test to verify whether there is a statistical difference between the two groups.

**Figure 2** shows the workflow of radiomics implementation. Before extracting the radiomics features, the original images were normalized through z score transformation, and the average intensity range for each imaging mode for all subjects was 0, with a standard deviation of 1. We used a two-step feature selection method to reduce curse of dimensionality, minimize overfitting, and determine the most effective feature for distinguishing PHEO and LPA. Firstly, a single factor analysis of variance (ANOVA) was performed on all features with ICC scores>0.75, selecting statistically significant features for the training cohort. Secondly, the selected features are included in the Least Absolute Shrinkage and Selection Operator (LASSO) regression algorithm to determine the best feature (with non zero coefficients) to distinguish between PHEO and LPA (**Figures 3A–C**). Tuning regularization parameters that control regularization intensity were selected by using a minimum standard of 10 times cross validation λ. Then, the final selected feature with a nonzero coefficient was used to construct a radiomics signature. Features weighted by their corresponding nonzero coefficients were screened using a linear combination of selected values. Then, the Rad scores of each patient in the training cohort and external validation cohort were calculated (**Figure 4**).

### Development of the radiomics nomogram and assessment of the performance of different models

Integrate independent clinical factors and Rad scores developed on the training cohort into the radiomics nomogram using multivariate logistic regression. Then, the Rad score and independent clinical factors were used to calculate the radiomics nomogram score (Nomo score) for each patient in the training and validation cohort.Calibration curves for two groups of patients were graphically generated to evaluate the performance of the radiomics nomogram. The AUC, accuracy, specificity, and sensitivity were used to evaluate the effectiveness of different models. The calibration efficiency of the nomogram was evaluated using a calibration curve, and the analysis fitting was performed using the Hosmer Lime test, which was used to evaluate the calibration ability of the nomogram. Decision curve analysis (DCA) was used to evaluate the clinical application value of the prediction model. The DeLong test was used to evaluate the difference in prediction efficiency between different models.

### Statistical analysis

Statistical analysis was conducted using R software (version 4.2.0, https://www.R-project.org). Qualitative data analysis was conducted using Fisher’s exact test or chi-square test, and quantitative data analysis was conducted using independent sample t-test. Delong test was used for comparing the predictive value of different models.The following software packages were used in our study: use the “glmnet (R)” software package for LASSO regression based on multivariate binary logistic regression. The ROC curve was plotted using the software package ‘Partial Subject Operating Characteristics (pROC [R])’. Use the ‘Regression Modeling Strategy (rms [R])’ software package for nomogram development and calibration curves. The significance level is set at p<0.05.

## Results

### Clinical factors of the patients

Comparison of clinical data and CT image characteristics of all patients with PHEO and LPA showed statistically significant differences (P< 0.01) in lesion location, maximum lesion diameter, necrosis, edge, CT values and perfusion values, as shown in **Table 2**.

**Table 2:** Clinical factors of the patients.

Clinical factors		PHEO(n=165, 184 lesions)	LPA (n=167, 168 lesions)	*p-value*	χ^2^ or t
**Gender**	Male	73(44.2%)	62(37.1%)	0.19	1.74*
	Female	92(55.8%)	105(62.9%)		
**Age, year**		51.3 ± 12.6	51.2 ± 12.7	0.95	0.07
**Location**	left	83(50.3%)	102(61.1%)	<0.01	16.17*
	right	65(39.4%)	64(38.3%)		
	bilateral	17(10.3%)	1(0.6%)		
**Maximum diameter(mm)**		49.4 ± 28.3	23.5 ± 11.8	<0.01	11.02
**Hypertension**	positive	88(53.3%)	97(58.1%)	0.66	0.19*
	negative	77(46.7%)	70(41.9%)		
**Necrotic**	positive	138(75.0%)	47(28.0%)	<0.01	77.88*
	negative	46(25.0%)	121(72.0%)		
**edge**	positive	115(62.5%)	154(91.7%)	<0.01	41.46*
	negative	69(37.5%)	14(8.3%)		
**Arterial phase CT value(CT_A) (Hu)**		123.2 ± 51.0	71.8 ± 25.5	<0.01	11.79
**Conventional CT value (Hu)**		41.4 ± 7.1	27.1 ± 9.3	<0.01	16.32
**Perfusion value (Hu)**		81.8 ± 52.4	44.6 ± 22.2	<0.01	8.50

* **χ^2^** test, Perfusion value=Arterial phase CT value-Conventional CT value.

### Validation of the models

**Figure 5:**
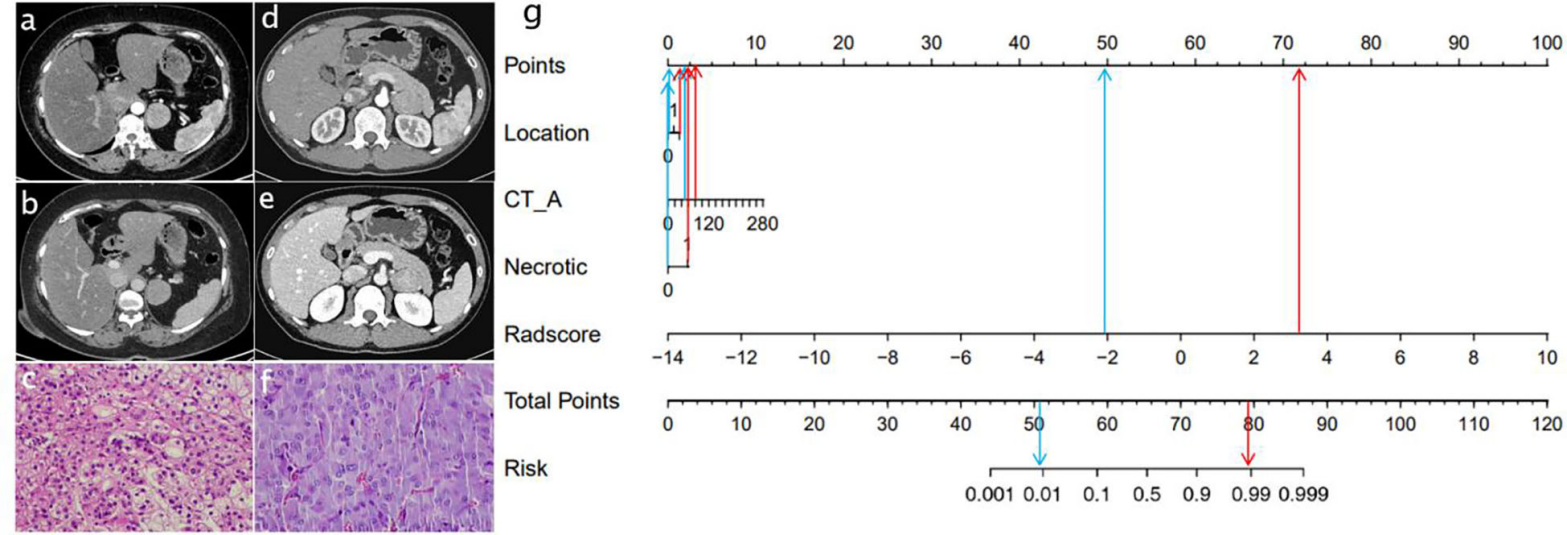
Radiomics nomogram used to classify LPA and PHEO.(CT_A: Arterial phase CT value) Data of a patient with LPA **(A–C)**,data of a patient with PHEO **(D–F)**; radiomics nomogram **(G)**. The lesions of two patients had similar imaging findings.

**Figure 6:**
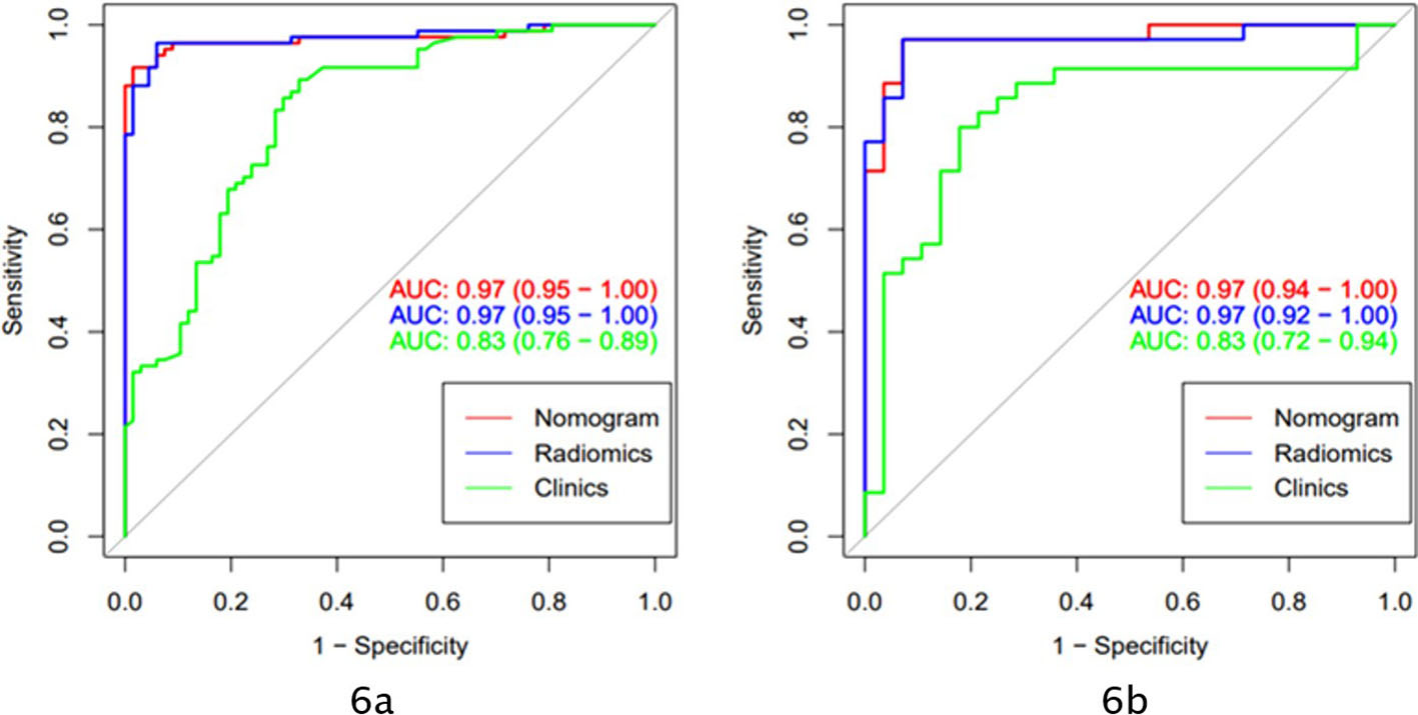
The models based on clinical and conventional CT scan images applied AUC to evaluate the prediction ability of different models (**A**: ROC curve of the training cohort; **B**: ROC curve of the validation cohort).

**Figure 7:**
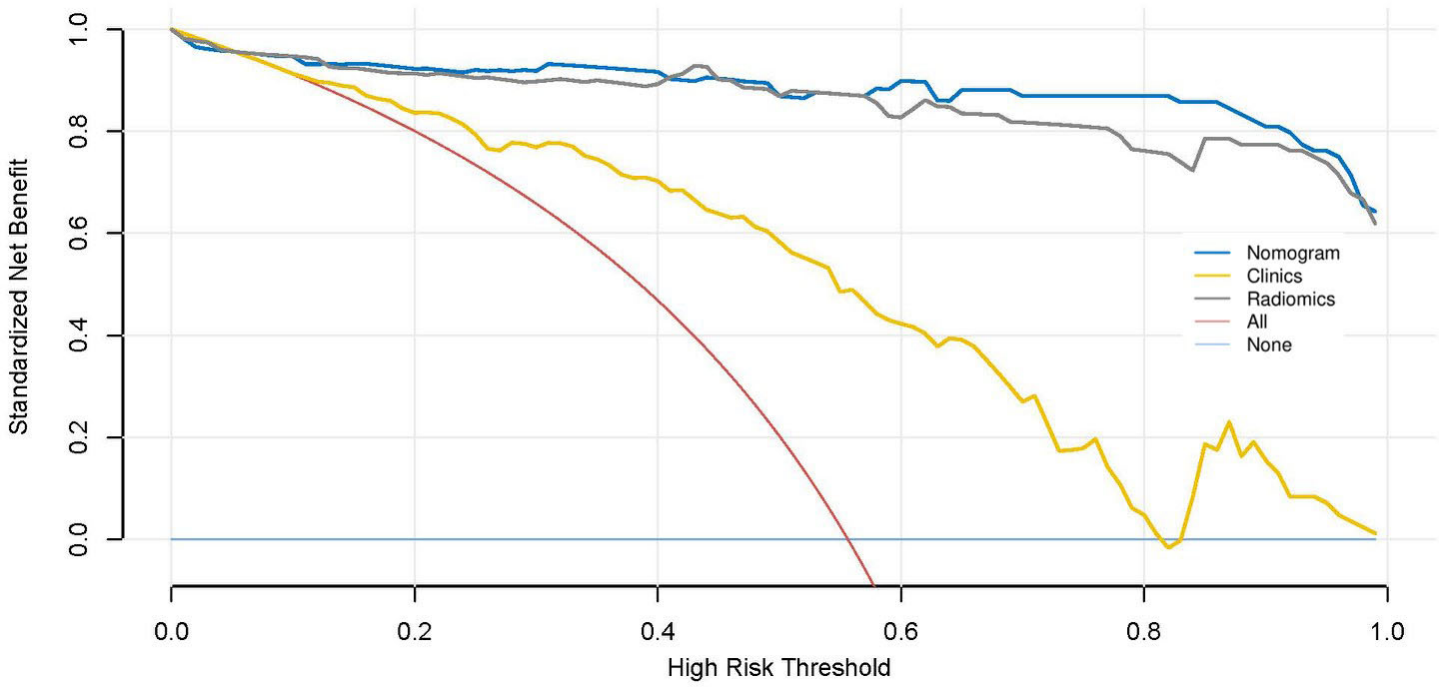
Decision curve analysis (DCA) of different models.

The AUC of the clinical model was 0.83 (95% CI: 0.76-0.89) in the training cohort and 0.83 (95% CI: 0.72-0.94) in the validation cohort. Clinically relevant factors of lesion location, CT values (arterial phase CT values), and necrosis were independent predictors for classifying PHEO and adrenal LPA, and these factors were integrated with the radscore to create a nomogram, as shown in **Figure 5**. The predictive effectiveness of the clinic model,radiomics signatures and radiomics nomogram models established based on conventional CT images and dynamic enhanced CT images (Mixed images of arterial and venous phases) are shown in **Table 3**. We compared the predictive value of different models using Delong test, and the results showed that the prediction efficiency of the model based on enhanced CT images was slightly higher than that based on conventional CT images, but the difference was not statistically significant (p>0.05)(**Table 3**). **Figure 6** shows that in the validation cohort, the predictive ability of the radiomics nomogram (AUC=0.97, 95% CI: 0.94-1.00) and radiomics signature (AUC=0.97, 95% CI: 0.92-1.00) based on conventional CT images was better than that of the clinical model (AUC=0.83, 95% CI: 0.72-0.94). **Figure 7** shows the DCAs of the radiomics nomogram and radiomics signature.

**Table 3:** Comparing the predictive value of different models using Delong test.

Models	Training cohort				*p*- value	Validation cohort				*p*- value
	AUC (95% CI)	Accuracy	Sensitivity	Specificity		AUC (95% CI)	Accuracy	Sensitivity	Specificity	
CT-conventional radiomics signature	0.97 (0.95~1.00)	0.95	0.96	0.94	Reference	0.97 (0.92~1.00)	0.92	0.82	0.86	Reference
Clinical model	0.83 (0.76~0.89)	0.79	0.89	0.67	<0.001	0.83 (0.72~0.94)	0.68	0.91	0.39	0.03
Enhanced CT radiomics signature	0.98 (0.97~1.00)	0.95	0.94	0.96	0.94	0.98 (0.95~1.00)	0.89	0.97	0.79	0.48
CT- conventional radiomics nomogram	0.97 (0.95~1.00)	0.95	0.92	0.99	0.98	0.97 (0.94~1.00)	0.91	0.87	0.96	0.74
Enhanced CT radiomics nomogram	0.98 (0.97~1.00)	0.96	0.98	0.94	0.87	0.97 (0.94~1.00)	0.89	0.85	0.96	0.81

p-value: Comparing the predictive value of different subgroup models using Delong test.

Both radiomics models and clinical models can predict the classification of PHEO and LPA. In the training and validation cohorts, the predictive ability of the nomogram (red) (AUC=0.97) and the radiomics signature (blue) (AUC=0.97) were better than that of the clinical model (green) (AUC=0.83).

Net income is displayed on the y-axis. The dark blue line represents the nomogram model model that combined clinical features and radscores. The red line indicates that all patients are assumed to have PHEO, the light blue line indicates that none of the patients have PHEO, the yellow line represents the results of the clinical model, and the gray line represents the results of radiomics signature. It can be seen that the radiomics signature model and the nomogram model have higher net income.

## Discussion

Adrenal adenoma is very common in clinical work, and CT has high specificity and sensitivity in the diagnosis of adrenal adenoma when its CT value on conventional scan is below 10 Hu due to its rich lipid component. However, some adrenal adenomas lacking lipids (called LPAs) are difficult to diagnose correctly (11, 12). PHEOs can secrete catecholamines. The typical clinical manifestation is hypertension, as well as headache and palpitations associated with hypertension, but in practice, approximately 10% to 20% of patients have no clinical manifestations or atypical manifestations (13, 14). Adrenal LPA and PHEO are both blood-rich tumors and have many similarities in CT presentation that make them difficult to differentiate (15–18). However, the surgical risk of PHEO is high, and the literature reports that adequate preoperative preparation could reduce the surgical mortality of PHEO from 30%-40% to 0-5.5% (19–21). Therefore, it is necessary to make an accurate clinical diagnosis of patients before surgery.

In the past, the relative and absolute enhancement washout rates were often used to characterize adrenal tumors, but Park said that it was difficult to identify PHEO and LPA using the enhancement washout rate (22, 23). In studying abdominal energy spectrum CT, Marin et al. found that lipid-rich components showed a certain pattern of CT value changes in a certain energy range with high specificity but had little diagnostic significance for lipid-poor components (24).

More and more radiomics analysis was being applied to medical imaging research (25). Radiomics can help clinicians make accurate diagnoses by exploring the connection between images and pathology and clinics (26–28). In addition, radiomics characteristics may be important predictive factors for cancer differential diagnosis, treatment response, and survival prediction (29, 30). Xiaoping Yi et al (31) found that non enhanced CT quantitative texture analysis based on machine learning may be a reliable quantitative method for distinguishing PHEO from LPA. However, the sample size of this study was relatively small, and no model based on enhanced scanning 3D data had been established for comparison. Therefore, our study is the first to establish multiple imaging radiomics models based on conventional CT and enhanced CT images to predict LPA and PHEO, and we also compared the predictive performance of different models. The AUCs of the radiomics signature based on conventional CT images were 0.97 in the training cohort and 0.97 in the validation cohort. In the validation cohort, the AUCs of the radiomics signature and radiomics nomogram based on enhanced CT images were 0.98 and 0.97, respectively. Both models showed good predictive ability, better than the predictive performance of the clinical model. These results are also superior to the findings of Xiaoping Yi. The radiomics nomogram based on conventional CT images also yielded satisfactory results. The Delong test results showed that the prediction efficiency of the models based on enhanced CT images was slightly higher than that of the models based on conventional CT images, but the difference was not statistically significant (P >0.05). CT scanning can cause ionizing radiation damage, and dynamic enhanced scanning not only increases radiation exposure but also produces harmful effects such as contrast agent allergy and contrast agent nephrotoxicity (32–35). At present, radiological examinations should strictly follow the principle of “As Low As Reasonably Achievable” (32, 33, 36–38). The model based on conventional CT scans can effectively distinguish adrenal LPAs from PHEOs, and the radiation and contrast hazards associated with further enhancement scans can be avoided.

In this study, radiomic features were selected to construct radiomics signature model for classifying PHEO and adrenal LPA,including:P_wavelet_LLH_gldm_Dependence Non Uniformity,P_wavelet LLH_glszm_Large Area Low Gray Level Emphasis,P_wavelet_HHH glrlm_Run Length Non Uniformity Normalized,P_wavelet_LLH_glrlm_Run Length Non Uniformity Normalized,P_original_shape_Sphericity,P_wavelet_LLH_glcm_Contrast,P_original_shape _Minor Axis Length, P_original_firstorder_Median,P_wavelet_LLH_glrlm_Run Variance, among which 1 first order feature, 3 glrlm features,1 glszm feature,1 glcm feature,1 gldm feature and 2 original shape features were included.A mix of first-order, texture and wavelet features seemed to be important for classifying PHEO and adrenal LPA. In our study, we used filters to extract radiomics features from the original images.Among the 9 independent imaging features ultimately selected, there are 6 wavelet features. Wavelet features can comprehensively analyze changes in spatial frequency. These features can provide detailed analysis of texture changes. Wavelet features can also quantify the heterogeneity of tumors in various directions through different spatial scales, so it is believed that wavelet features may help us understand the pathophysiology and morphology of tumors (39). Previous studies had revealed the potential value of wavelet features in histological subtype prediction and prognostic assessment (40, 41). Our results show that wavelet features also have significant capabilities in the prediction models of PHEO and LPA.First order features can reflect the grayscale distribution of tumors and are obtained by calculating the grayscale values of tumors, usually representing low dimensional information that is easy to perceive visually.In addition, our model also includes two original shape features, which respectively suggest that the short axis length and sphericity of the tumor may have value in distinguishing PHEO and LPA.

Nevertheless, our research has some limitations: (1) there may be problems of selection bias and information bias in retrospective studies; (2) different CT machines reduce the consistency of image comparison to a certain extent; and (3) future multicenter and prospective trials are needed to verify the results of this study.

In conclusion, the CT-based radiomics signature and radiomics nomogram in our research have good predictive efficacy in identifying PHEO and adrenal LPA. The model based on conventional CT scans can identify both diseases while avoiding the radiation and contrast hazards caused by dynamic enhancement scans.

## Data Availability

The original contributions presented in the study are included in the article/supplementary material, further inquiries can be directed to the corresponding author/s.
